# Detection of 2-Furaldehyde in Milk by MIP-Based POF Chips Combined with an SPR-POF Sensor

**DOI:** 10.3390/s22218289

**Published:** 2022-10-28

**Authors:** Giancarla Alberti, Francesco Arcadio, Maria Pesavento, Chiara Marzano, Luigi Zeni, Naji Abi Zeid, Nunzio Cennamo

**Affiliations:** 1 Department of Chemistry, University of Pavia, Via Taramelli n.12, 27100 Pavia, Italy; 2 Department of Engineering, University of Campania Luigi Vanvitelli, Via Roma n.29, 81031 Aversa, Italy; 3 Industrial Research Institute, Lebanese University Campus Hadath, Baabda 2806, Lebanon

**Keywords:** optical-chemical sensors, 2-furaldehyde (2-FAL), milk, plastic optical fibers (POFs), molecularly imprinted polymers (MIPs), surface plasmon resonance (SPR)

## Abstract

An innovative optical-chemical sensor has been used to detect the 2-furaldehyde (2-FAL) in milk. The proposed sensing approach exploits the refractive index changing in a microstructured chip based on a plastic optical fiber (POF) with orthogonal micro-holes containing a specific molecularly imprinted polymer (MIP). This POF-MIP chemical chip modifies the surface plasmon resonance (SPR) phenomena excited in another sensor chip realized in POFs (SPR-POF) and connected in series. The proposed sensor configuration exploits MIP receptors avoiding any modification of the gold film of the SPR platform. This work reports the performance, particularly the high sensitivity and low detection limit, in complex matrices such as buffalo milk fortified with 2-FAL and in different commercial kinds of cow milk thermally treated for pasteurization. The measurements were carried out in about ten minutes by dropping the solution under-test on the planar D-shaped POF surface of the chemical chip. In contrast, on the gold surface of the SPR-POF platform, a water drop is always placed to excite the SPR phenomenon, which is modulated by the chemical chip via MIP-2-FAL binding. Furthermore, the experimental results demonstrated the pros and cons of the proposed sensor system. Thanks to the high sensitivity of the sensor system, the detection of 2-FAL in the diluted milk sample (1:50) was achieved. The dilution is required to reduce the interferent effect of the complex matrix.

## 1. Introduction

Dairy products, particularly milk, have a significant role in individual nutrition, especially for infants and children. Heat treatments are the generally employed processing technology used for drying and sterilization; sterilization allows for keeping milk at room temperature for months. Milk can be pasteurized at ultra-high temperature (UHT) and bottled sterilization. Unfortunately, these processes give rise to by-products, flavors, and unpleasant colors. For example, Maillard’s reaction can produce various toxic substances. Furfurals are produced at the intermediate stage of Maillard’s reaction, and they can help assess how heat treatments lead to a harmful alteration of the milk [1]. Indeed, by subjecting the milk to storage at inadequate temperatures or heat treatment, several furfural derivatives, such as 2-furaldehyde, 5-hydroxymethylfuraldehyde 2-furylmethylcetone, and 5-methyl-2-furfural, can be produced [1,2]. Toxicological studies highlighted furfurals’ possible mutagenic and genotoxic effects [3,4]. These substances can adversely affect the liver, kidney, human central nervous system, or other organs at concentrations higher than the maximum body’s adsorption limit [5].

Consequently, developing sensitive and accurate methods to detect furfurals in milk is essential. Classical analyses are based on chromatographic or spectrophotometric techniques [6,7,8,9,10,11,12,13,14,15,16]. Chromatographic methods are undoubtedly the most common, but some of them have scarce specificity; moreover, the instruments are bulky and require toxic reagents and solvents. Spectrophotometric UV-vis methods are more straightforward and less expensive, but often they suffer from matrix effects, poor selectivity, and insufficient sensitivity for trace analyses. Thus, the need for fast, portable, low-cost methods and sensors for detecting furfurals compounds in dairy foods is crucial.

Currently, electrochemical and optical sensors have been proposed; most are devoted to determining furfurals in transformer oils and wastewaters [17,18,19,20,21,22,23,24,25], and few detect these compounds in dairy products. For example, Rong et al. [26] developed a fluorimetric nanosensor to quantify 2-furaldehyde (2-FAL) in different foods, including milk. The method was accurate but not sensitive enough for detecting 2-FAL at trace levels; moreover, it required samples’ pretreatment and toxic reagents.

A disposable colorimetric device based on a polymeric film was developed for sensing furfurals in beer, measuring the red-green-blue (RGB) color coordinates of the polymeric film after exposure to the sample by a smartphone [27]. However, the good analytical figures of merit and the limit of detection (LOD) of about 12 µg/L make it inapplicable to not contaminated samples.

An electrochemical sensor, based on reduced graphite oxide for voltammetric determination of 2-FAL in milk products, was proposed by Wang Q. et al. [28]. Despite a wide linear concentration range and pretty good precision, a lengthy pretreatment of the sample was required, and the LOD, even in this case, is inadequate for quantifying 2-FAL in not fortified samples.

Recently, colorimetric paper-based test strips were developed for sensing 2-FAL and 5-hydroxymethylfurfural (5-HMF) in fruit juices, obtaining good performance in terms of selectivity but allowing only a semi-quantitative measure [29].

Fiber optic chemo and biosensors are well-suitable for different applications, including food analysis [30,31]; particularly, those based on surface plasmon resonance (SPR) [32,33,34,35] allow the detection of several analytes with high sensitivity. A further step forward in this field was also made by introducing plastic optical fibers (POFs), allowing the realization of small platforms integrable with pocket-sized optoelectronic devices [33].

SPR phenomenon occurs when a polarized light beam causes the excitation of free electrons’ oscillations (surface plasmon wave) at a noble metal nanofilm’s surface-dielectric interface. When the energy and the momentum of the incident light and the surface plasmon wave match, a resonance happens, resulting in a sharp drop in the intensity of the reflected light [34]. The resonance strictly depends on the refractive index of the dielectric surrounding the metallic nanofilm, so any change in the refractive index of the dielectric produces a variation in the propagation of the surface plasmon wave, and this change can be used for analytes sensing [36]. The SPR-POF sensors’ sensitivity can be increased by modifying their geometry and adding high refractive index layers. Different plasmonic sensor configurations with ultra-high performances useful to monitor refractive index variations in contact with the plasmonic sensitive area have been recently presented [37,38]. In these plasmonic probes, the selectivity can be obtained by coupling the plasmonic area with molecular recognition elements (MREs) of biological (enzymes, antibodies, aptamers) or synthetic nature, such as molecularly imprinted polymers (MIPs) [33].

Our research group developed a MIP-based SPR-POF platform for detecting 2-FAL in wine [39]. In that case, the SPR phenomenon occurred at a gold-photoresist multilayer structure obtained from a planar surface of exposed core POF inserted in a resin block (D-shaped platform), and the MIP layer was placed on the multilayer. However, with this kind of D-shaped platform, a crucial point is the thickness of the MIP layer. Indeed, with a media with a relatively low refractive index (i.e., water), high sensitivity can be achieved with a thinner MIP layer; conversely, with matrices at a high refractive index (for example, transformer oil), a high thickness is desired for avoiding a matrix bulk effect [40].

Aiming to solve these problems, we have recently proposed a novel platform configuration in which the MIP is deposited in micro-holes drilled into the exposed core of a micro-structured D-shaped POF (MIP-POF3). The variation of the waveguide structure upon interaction with the analyte in the micro-hole was evaluated by the SPR phenomenon excited via the previously described D-shaped POF platform. The measurements are, therefore, not affected by the MIP thickness or bulk effects. The device was applied to detect 2-FAL in standard aqueous solutions obtaining ultra-low detection limits and good selectivity [25].

Given the excellent results achieved in aqueous solutions, in order to test the performances of the new device in a very complex matrix, in the present paper, the same platform is applied to a direct determination of 2-FAL in milk samples. The results indicate that the 2-FAL detection can be achieved by reducing the pretreatments and decreasing the analysis time and cost (usually required for standard laboratory tests). In particular, we demonstrate that only one dilution step of the milk samples is necessary, and a few minutes of incubation are sufficient for reliable measurements.

The validity of the proposed method was assessed by analyzing commercial UHT milk samples and comparing the results with those obtained by GC-MS as a standard method.

## 2. Materials and Methods

### 2.1. Reagents and Materials

2-furaldehyde (2-FAL), Divynilbenzene (DVB), Methacrylic acid (MAA), 2,2′-azobisisobutyronitrile (AIBN), from Merk Life Science S.r.l. (Milano, Italy), were employed. The stabilizers of DVB and MAA were removed by SPE (solid phase extraction) on a glass cartridge filled with alumina (Merk Life Science S.r.l. (Milano, Italy). All other reagents were used as received.

The pasteurized milk samples (milkCP) and UHT (milkCUHT) Berna–Parmalat S.p.A. (Milano, Italy) were purchased in a local supermarket (Aversa, Italy). The untreated buffalo milk (milkB) was obtained from a local firm (Grazzanise (Caserta), Italy). The 2-FAL concentration in these samples has been determined by a reference method.

### 2.2. Prepolymeric Mixtures for MIP

MIP’s prepolymeric mixture was prepared according to the previously described procedure [39,40,41]; it consisted of 2-FAL, MAA, and DVB in the molar ratio of 1:4:40. DVB acted as crosslinker and solvent. The mixture was sonicated and deaerated with a gentle flow of nitrogen for ten minutes; then, an excess of AIBN was added as a radical initiator.

The MIP synthesized from this prepolymeric mixture was previously applied to different optical and electrochemical sensors [39,40,41] and characterized by Scanning Electron Microscopy (SEM), Fourier-transform infrared (FT-IR), and Differential Scanning Calorimetry (DSC) analyses [41] confirming the successful molecular imprinting. The MIP for 2-FAL was synthesized by a typical non-covalent approach, and the template recognition occurs through weak interactions and H-bonds.

### 2.3. Optical-Chemical Sensor System and Procedure for the Measurement of 2-FAL

**Figure 1:**
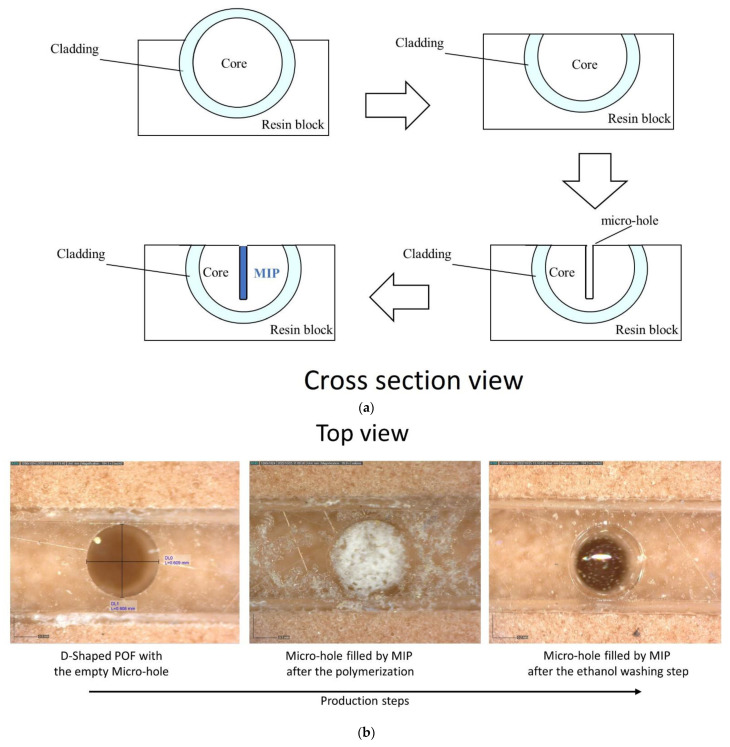

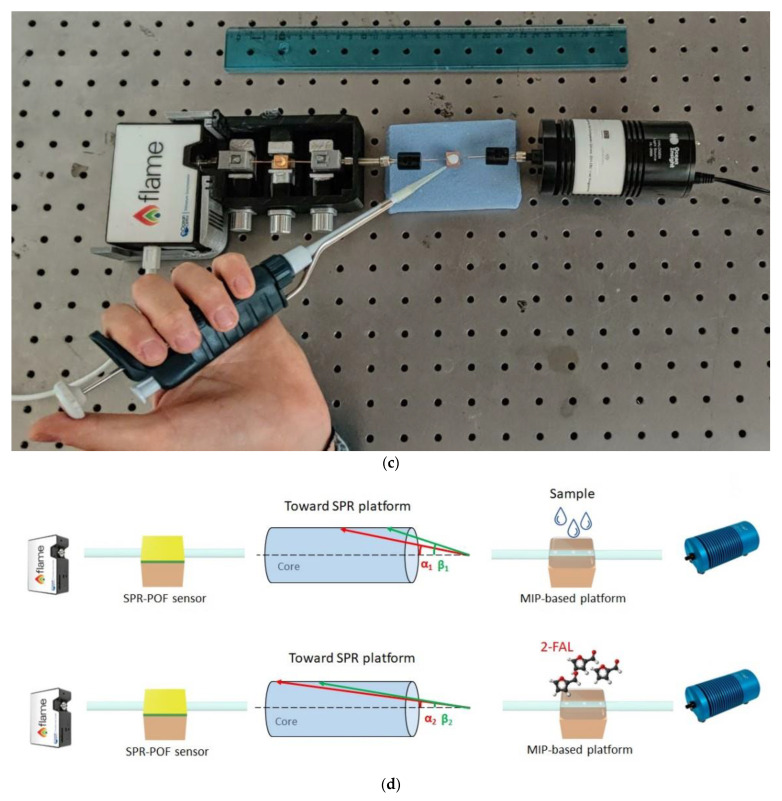
(**a**) Outline of the chemical chip manufacturing steps. (**b**) Optical images of the chemical sensing surface in the production steps. (**c**) Experimental setup showing the equipment, the SPR-POF platform, and the MIP-POF3 chip. (**d**) Sensing principle of the optical-chemical sensor system.

A sensing approach recently developed by our research group [25] is used to measure the analyte of interest (2-FAL) in milk. The proposed sensing methodology exploits the effect of the change in the mode profile of the light in a modified multimode POF chip (chemical sensor chip) that interacts with the analyte of interest, connected at the input of an SPR-POF sensor [42]. The experimental setup is characterized by a halogen lamp, illuminating the chemical chip, an SPR-POF platform, and a spectrometer, as shown in Figure 1. The employed halogen lamp exhibits a wavelength emission range from 360 nm to 1700 nm (model HL2000-LL, manufactured by Ocean Insight, Orlando, FL, USA), while the spectrum analyzer detection range was from 350 nm to 1000 nm (model flame, manufactured by Ocean Insight, Orlando, FL, USA).

The chemical sensor chip (MIP-POF3) is based on a D-shaped POF chip with three micro-holes filled with a prepolymeric mixture for MIP [25]. A 1 mm POF (980 μm PMMA core and 10 μm cladding) has been used to obtain the chemical sensor chip. Firstly, the POF was embedded in a resin block for the polishing process, obtained by two different polishing papers (5 μm and 1 μm grits), in order to realize the D-shaped POF region. The resin cube-shaped is 1 cm × 1 cm × 1 cm. Then, by taking advantage of a computer numerical control (CNC) micro-milling machine, three micro-holes (diameter of 600 μm) were achieved into the exposed core of the POF in an orthogonal orientation to the direction of the propagating light. The obtained micro-holes have been filled with the prepolymeric mixture, and a thermal polymerization step is then carried out by placing the platform in the oven at 80 °C for 16 h [25]. The template (2-FAL) was extracted from the MIP by repeated washing with 96% ethanol and finally with Milli-Q water. Figure 1a shows the cross-section view of an outline of these production steps, whereas Figure 1b reports the images acquired by the Dino-Lite digital microscope (AnMo Electronics Corporation, New Taipei City, Taiwan) relative to the chemical sensor chip in successive production steps.

On the other hand, the SPR-POF sensor is the same probe previously proposed and widely described [42], with a fixed liquid (water) on the gold layer (60 nm thick) surface. In this configuration, it is used to monitor the variation of the light modes taking place in the chemical chip [25]. Therefore, when the binding between the 2-FAL and the MIP occurs, the refractive index of the POF core in the chemical sensor changes along with the SPR phenomenon in the SPR-POF sensor [25]. In particular, the resonance wavelength shifts towards lower wavelengths (blue shift) when the 2-FAL concentration increases by increasing the MIP refractive index in the core of the POF.

A picture of the sensor system is shown in Figure 1c, while the principle of sensing is illustrated in Figure 1d. The binding 2-FAL-MIP causes a change in incidence angles associated with each propagated mode in the SPR-POF sensor by changing the SPR condition (such as the resonance wavelength used in these measurements) [25], as shown in Figure 1d.

The binding of 2-FAL to the MIP in the micro-hole has been evaluated using a protocol that consists in dropping 60 μL of the milk sample, fortified with a known amount of 2-FAL, upon the sensitive surface of the microstructured chemical platform and acquiring the spectra after ten minutes of incubation to enable the interaction between the analyte and the MIP in the holes to take place.

The SPR spectra were acquired in water after equilibration with the milk sample for 10 min, followed by a washing procedure usually performed by flushing with water 20 times and one static washing with a drop of water for 5 min.

The spectra were always acquired with water over the SPR platform. The spectrum for the normalization was acquired with air over the SPR platform and water over the MIP platform.

The signal (Δλ) was the variation of the resonance wavelength taking place when the target combines with the MIP in the micro-holes, so producing a local variation of the refractive index of the waveguide, compared to that of a blank sample, i.e., with the same composition of the sample under investigation but not containing 2-FAL [33]. MilkB was assumed as a blank solution for all the considered milk samples.

The dose–response curves were obtained by plotting Δλ vs. *c*, where *c* is the analyte concentration. The experimental points were fitted by the Hill model, as previously described [25].

## 3. Results

The performance of the proposed optical-chemical sensor system in milk has been investigated by the dose–response curves obtained by measuring the resonance wavelength of the SPR platform in water at increasing 2-FAL concentrations in standard solutions prepared in fresh milk. In particular, untreated buffalo milk (milkB) was considered for this investigation since it is believed to have a negligible content of furanic compounds, which, on the other end, increases after treatments at high temperatures, as previously established by classical chromatographic analyses [6,7,8,9,10,11,12].

At the same time, the matrix composition is similar to that of other milk samples, so it can be used as a blank.

The same optical-chemical sensor system proposed for determining 2-FAL in water [25] has been tested in milk, which is a much more complex matrix with different biochemical properties and a diverse bulk refractive index (RI of milk ranges from 1.34 to 1.35).

**Figure 2:**
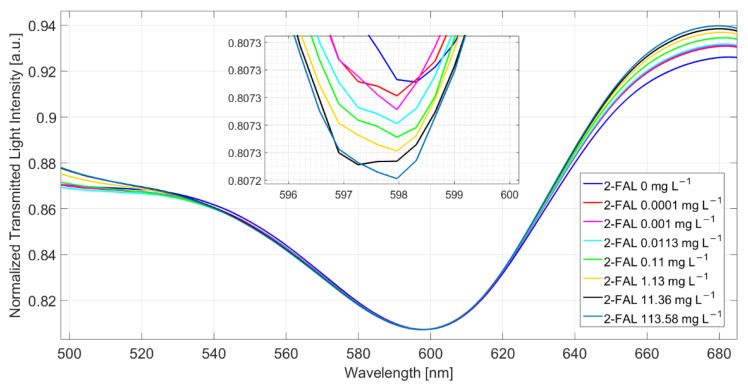
SPR spectra at different 2-FAL concentrations measured directly in buffalo milk after 10 min of incubation.

Figure 2 shows the spectra obtained at different 2-FAL concentrations directly in the fresh buffalo milk (milkB) after 10 min of incubation. The reference spectrum for the normalization is acquired when air is the surrounding medium on the SPR-POF chip (in this case, the SPR condition is not satisfied) and water on the MIP-based chip. During all measurements, a medium with a fixed refractive index equal to 1.332 (water) is present on the SPR-POF chip [25].

A slight shift toward lower resonance wavelengths (blue-shift) of about 0.2 nm is observed when the sample is fortified with 113.58 mg/L of 2-FAL. This behavior suggests that some substances are adsorbed by the MIP from the milk matrix, hindering the template’s interaction with the imprinted sites.

**Figure 3:**
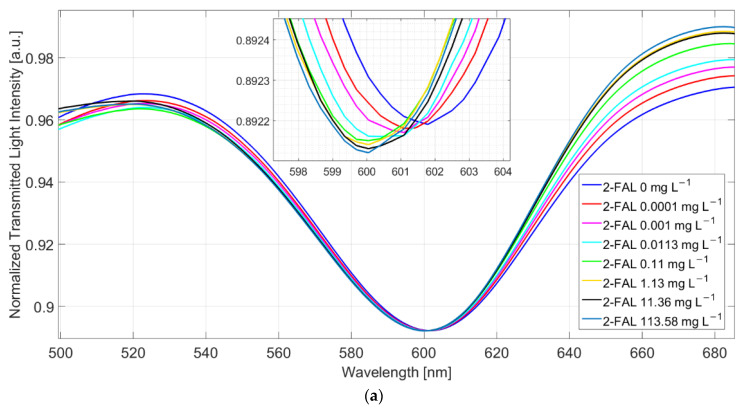

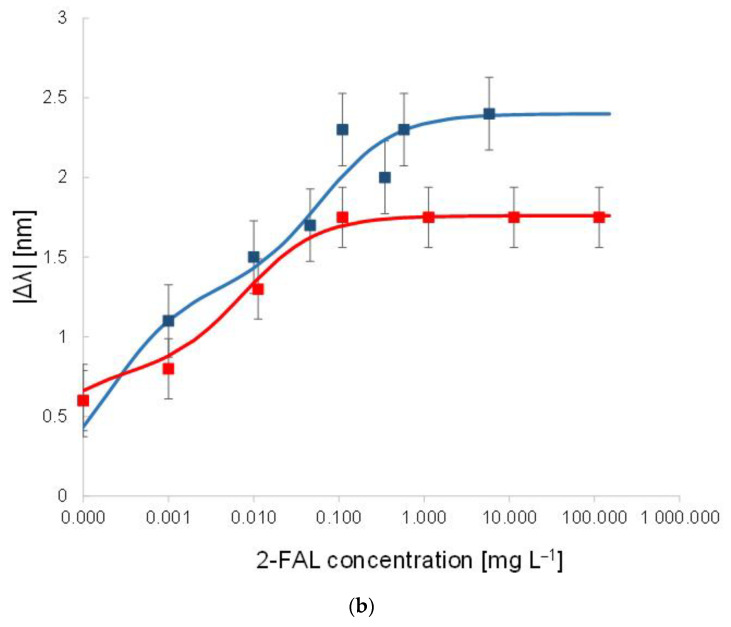
(**a**) SPR spectra at different 2-FAL concentrations in untreated buffalo milk, measured after 10 min of incubation and a washing procedure. (**b**) Dose–response curve of 2-FAL in buffalo milk (red points) and aqueous solutions (blue points). Continuous curves are the best fitting obtained by the two sites Hill model (Equation (1)).

For this reason, all subsequent measurements were made after removing the substances unspecifically adsorbed by repeated washing with water, as described in the experimental part. Figure 3a reports the SPR spectra, normalized to the reference one, of standard solutions at different concentrations of 2-FAL in milkB obtained in this way.

At increasing furfural concentration, the resonance wavelength shifts toward lower values (blue-shift). This behaviour is in accordance with that observed in [25], where both numerical and experimental results denoted that, for a local increase in the core’s refractive index, the SPR wavelength decreases. The dose–response curve is reported in Figure 3b and compared with that previously obtained with standard aqueous solutions [25].

The proposed sensor system is robust in terms of reproducibility. In particular, the chemical response of ten chemical chips, combined with the same SPR-POF platform, are the same in terms of sensitivity at low concentrations, the limit of detection, affinity constant, etc. This aspect is a key novelty of the proposed approach, even if the results are produced by hand. When the chemical chip changes, a slight variation could occur in the resonance wavelength at the SPR-POF chip, so each sample is analyzed after a calibration is performed on the same platform. After testing ten similar chemical chips, by carrying out three times similar tests for each one (exploiting a good regeneration step), the maximum value of standard deviation experimentally obtained is about 0.15 nm, as reported in the error bars of the dose–response curve shown in Figure 3b.

The curve in milkB is similar to the one in water, within the error of the experimental points, indicating that the washing procedure is effective, i.e., not any substance able to interfere persists in the MIP after the washing procedure.

In the case of water, the shape of the dose–response curve has been ascribed to the presence of two different kinds of sites with different affinity [25]. By assuming the same model for the milk sample after washing (two sites Hill model), reported in Equation (1), the results in Table 1 have been obtained. The values for the first kind of sites are approximate since only a few points are useful for the evaluation.

(1) $$\left|\Delta \lambda \right|=\left|\Delta \lambda_{\max1}\right|\cdot \frac{c}{K_{1}+c}+\left|\Delta \lambda_{\max2}\right|\cdot \frac{c}{K_{2}+c}$$

The LOD is calculated by the following equation [25]:

(2) $$\mathrm{LOD}=\frac{3.3\cdot SE_{i}}{s_{\mathrm{low}\text{-}\mathrm{conc}}}$$

where *SE_i_* is the standard error of Δλ_max1_ and *s*_low-conc_ the sensitivity at low concentrations. The obtained LOD value is approximately 0.01 µg/L.

The two sites Hill equation (Equation (1)) is suitable for the characterization of the sensing device, but it is not convenient as a standardization curve. Nevertheless, it is seen that the experimental curve in Figure 3b can be reasonably approximated by a straight line at concentrations between 1 and 100 µg/L. The corresponding function is reported here (in parenthesis is the standard deviation of the parameter):

(3) $$\Delta \lambda =2.1(0.1)+0.39(0.04)\cdot logc_{\left(2-FAL\right)}\;\;R^{2}=0.974$$

This relationship can be convenient for quantification purposes in the given concentration range.

### 3.1. Study of the Interferences in the Milk Matrix

Two possible interfering substances have been considered, reinforcing the milkB matrix with high concentrations of them. One is 5-HMF, a furanic compound with a molecular structure similar to that of 2-FAL, which is also formed in milk or milk products, most often at concentrations higher than 2-FAL. The second one is bentazon, a widely used herbicide with a molecular structure completely different from the furanic compounds, but which could interfere by unspecific interaction with MIP.

**Figure 4:**
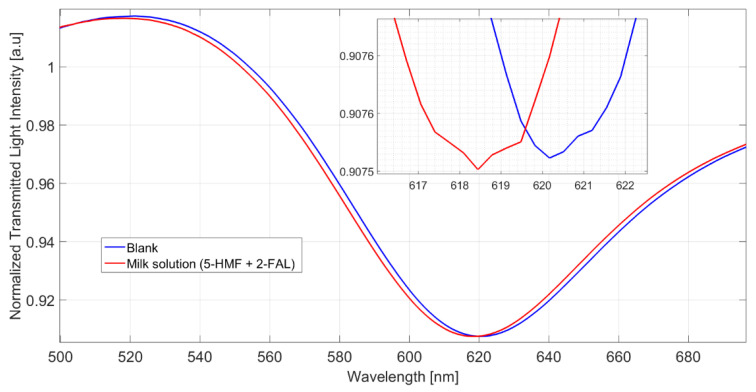
Spectra of milkB on POF-MIP3 fortified with 2-FAL 113.5 mg/L and 5-HMF 154 mg/L, measured after 10 min of incubation and a washing procedure.

The spectra obtained in a milkB sample fortified with 2-FAL and 5-HMF are reported in Figure 4.

**Figure 5:**
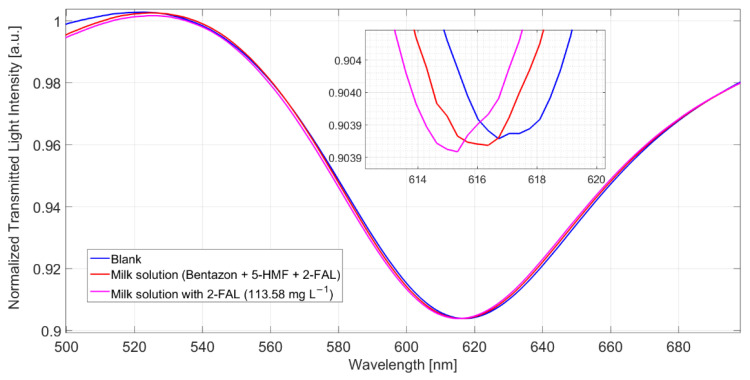
SPR spectra obtained by 5-HMF (154 mg/L), bentazon (105 mg/L) and 2-FAL (114 mg/L) in milkB, measured after 10 min of incubation and a washing procedure.

The shift referred to the blank solution is about 1.9 nm, which corresponds well to the saturation response to 2-FAL in the absence of the interfering substance; this shows that 5-HMF does not interfere in the determination of 2-FAL and demonstrates the high specificity of the imprinted sites in MIP. The spectra reported in Figure 5 were obtained in milkB fortified with 2-FAL and the two interfering substances, all at a concentration of about 100 mg/L. It can be observed that the blue shift referred to the blank solution is about 1 nm, much lower than that of the milk fortified with only 2-FAL at 113.58 mg/L (1.9 nm in this case), which is also reported in Figure 5 for comparison. Thus, the observed interference must be due to bentazon.

**Figure 6:**
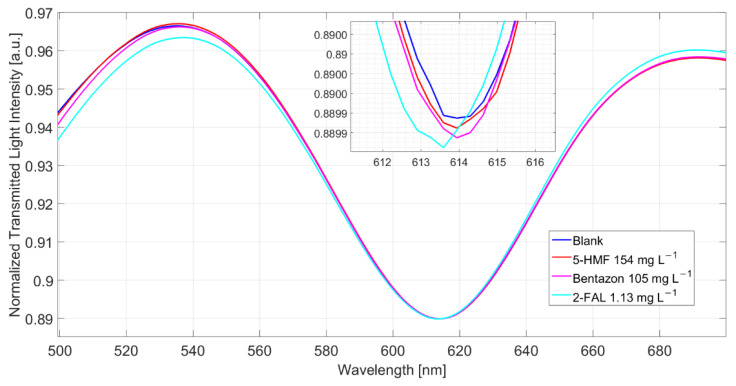
Spectra of milkB fortified with possible interfering substances, 5-HMF and bentazon, and comparison with the spectrum of 2-FAL at a similar concentration (the spectra are obtained after 10 min of incubation and a washing procedure).

The spectra of milkB, obtained in a different experiment, i.e., registered in samples separately reinforced with the two possibly interfering substances, are reported in Figure 6. The λ_res_ of the sample with 5-HMF and that with bentazon are similar to those in the not fortified milkB, indicating that both the substances do not produce any response, even when present at a high concentration. Nevertheless, it has been previously shown (see above) that bentazon interferes. Actually, the response to 1.13 mg/L of 2-FAL, after the measurements of milkB with the two substances, is lower than that expected from the dose–response curve reported in Figure 3b, being only 0.3 nm.

Notice that a similar effect was also observed in the case of water [25]. In that case, a milkB sample fortified with 1.13 mg/L of 2-FAL was measured after the experiments with the same sample spiked with 5-HMF and bentazon at 154 and 105 mg/L, respectively. A shift of 1.3 nm was measured, while in a sensor not previously contaminated with the interfering substances, the same concentration in water gave a higher Δλ, 2.2 nm.

A possible explanation could be that bentazon can interact with the MIP present in the micro-holes in POF but only with that located at the surface of the micro-holes so that no variation of the resonance wavelength is produced. Moreover, bentazon is not removed by the usual washing procedure, and its presence prevents the interaction of 2-FAL with the MIP’s imprinted sites inside the micro-holes.

### 3.2. Determination of 2-FAL in Milk

In order to test the performances of the proposed sensor system to identify 2-FAL in real samples, two commercially available kinds of milk treated at high temperatures were examined, cow milk pasteurized (milkCP) and cow milk UHT (milkCUHT).

The concentrations of 2-FAL in these two samples, determined by the GC-MS method [43], are 16(2) µg/L for milkCP and 22(6) µg/L for milkCUHT.

**Figure 7:**
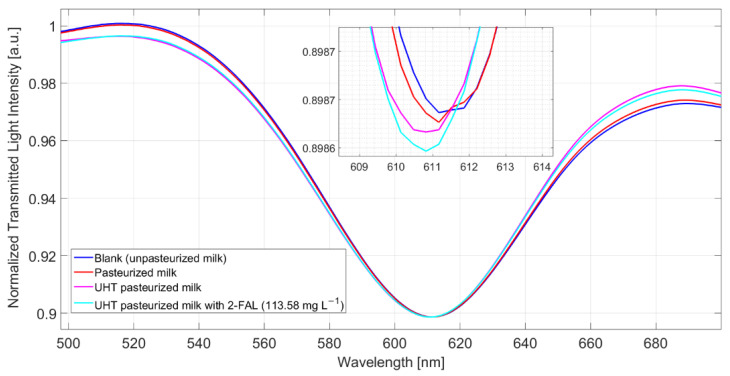
Spectra obtained from different kinds of milk (pasteurized milk, UHT milk, and UHT fortified with 2-FAL) measured after 10 min of incubation and a washing procedure.

MilkB, supposed not to contain furanic compounds, was considered as a blank solution, in reference to which Δλ was evaluated. Figure 7 shows the spectra obtained after 10 min of incubation and a washing procedure for milkB, milkCP, milkCUHT, and milkCUHT fortified with 2_FAL. As seen in the spectra reported in Figure 7, the pasteurized milk gave a modest resonance shift compared to the blank solution, while a more significant blue shift was observed for the UHT milk, 0.8 nm. In both cases, the concentration of 2-FAL evaluated in the pristine milk samples is much lower than that obtained by the GC-MS method. Moreover, adding a very high concentration of 2-FAL (113 mg/L) does not produce any significant variation of Δλ_res_ (see Figure 7). This indicates that some interfering substances must be present in these kinds of milk, i.e., cow milk thermally treated, which are not eliminated by the washing procedure successfully adopted for milkB. This effect is similar to that of bentazon reported above.

Taking advantage of the very high sensitivity of the proposed detection system, the two samples were highly diluted with water, 1:50, and measured with the same procedure used for the pristine samples. The experimental results reported in Figure 7 and those obtained by 1:50 diluted milk are summarized in Table 2, where the 2-FAL content has been evaluated by Equation (3).

The agreement with the concentration determined by the chromatographic method is good in the case of milkCUHT but not in milkCP. Actually, in this case, the Δλ_res_ measured is very near to the uncertainty of the measure of Δλ, as seen in Equation (2), where the significant signal would be 3.3 · *SE_i_* = 0.33 nm. In the case of milkCP, a lower dilution would have been considered to obtain a significant resonance wavelength shift.

## 4. Conclusions

The experimental results demonstrated that the proposed optical-chemical sensor system could be used to detect the 2-FAL in a very complex matrix as milk with a simple procedure, not requiring any sample pretreatments. The matrix influences the SPR spectra, so they must be registered in water after a simple washing procedure. Due to the high sensitivity of the method, a milk dilution with water is most often required.

The MIP-POF-based chemical chip is of simple realization; moreover, it is low-cost and can be re-used thanks to the easy regeneration of the MIP receptor, effectively obtained via vigorous ethanol washing. The obtained performances of the sensor system to detect 2-FAL in the untreated buffalo milk are very similar to those obtained in water solutions, demonstrating that the milk complex matrix does not strongly influence the measurements. However, this is not true in the case of thermally treated kinds of cow milk, which can be analyzed simply by diluting the sample with water, taking advantage of the high sensitivity and low detection limits of the proposed sensing device.

## Figures and Tables

**Table 1:** Parameters of the two sites Hill equation (Equation (1)) used for fitting the dose–response curve of 2-FAL in buffalo milk.

Δλ_max1_ [nm]		Δλ_max2_ [nm]		*K*_1_ [mg^−1^ L]		*K*_2_ [mg^−1^ L]		Statistics
Value	Std err	Value	Std err	Value	Std err	Value	Std err	Adj. R-square
0.8	0.2	0.9	0.2	1.0·10^−5^	0.3·10^−5^	8·10^−3^	2·10^−3^	0.98

**Table 2:** Concentration of 2-FAL in pristine and 1:50 diluted milk evaluated by Equation (3).

Sample	Δλ_res_ [nm]	2-FAL [µg L^−1^]
milkCUHT	0.8	0.5
milkCP	---	n.d.
milkCUHT dil. 1:50	0.8	26.2
milkCP dil. 1:50	0.4	24.5

## Data Availability

Not applicable.
